# Health Status After Cancer: Does It Matter Which Hospital You Belong To?

**DOI:** 10.1186/1472-6963-10-204

**Published:** 2010-07-13

**Authors:** Jon H Fiva, Torbjørn Hægeland, Marte Rønning

**Affiliations:** 1 Department of Economics, University of Oslo, PB 1095 Blindern, 0317 Oslo, Norway; 2 Statistics Norway, Research Department, PB 8131 Dep., 0033 Oslo, Norway

## Abstract

**Background:**

Survival rates are widely used to compare the quality of cancer care. However, the extent to which cancer survivors regain full physical or cognitive functioning is not captured by this statistic. To address this concern we introduce post-diagnosis employment as a supplemental measure of the quality of cancer care.

**Methods:**

This study is based on individual level data from the Norwegian Cancer Registry (n = 46,720) linked with data on labor market outcomes and socioeconomic status from Statistics Norway. We study variation across Norwegian hospital catchment areas (n = 55) with respect to survival and employment five years after cancer diagnosis. To handle the selection problem, we exploit the fact that cancer patients in Norway (until 2001) have been allocated to local hospitals based on their place of residence.

**Results:**

We document substantial differences across catchment areas with respect to patients' post-diagnosis employment rates. Conventional quality indicators based on survival rates indicate smaller differences. The two sets of indicators are only moderately correlated.

**Conclusions:**

This analysis shows that indicators based on survival and post-diagnosis employment may capture different parts of the health status distribution, and that using only one of them to capture quality of care may be insufficient.

## Background

Health care expenditures have grown dramatically over time in most industrialized countries, and currently account for on average 9 percent of gross domestic product across member countries in the Organization for Economic Co-operation and Development [1]. Evaluation of the use of these resources is important from both efficiency and equity perspectives. This requires relevant and reliable measures of quality of care.

Most comparative studies of serious illnesses such as cancer and pneumonia rely on survival rates as proxies for successful treatment (e.g. [1,2]). In many circumstances, however, survival rates may miss important aspects of the quality of care. Quality indicators for health institutions reflecting patients' long-term health status should therefore also be considered.

Several studies document that cancer may have negative effects on employment and the ability to work (e.g. [3-6]). A meta-analysis by de Boer et al. [7] shows that patients often regard returning to work as an indicator of complete recovery, and that employment is associated with a higher quality of life. In this study, we therefore introduce post-diagnosis employment as an alternative measure of successful cancer treatment. The main contribution of our study is to compare quality indicators based on post-diagnosis employment to quality indicators based on survival. To the best of our knowledge, such an analysis has not previously been undertaken.

A majority of the studies assessing quality differences across health institutions perform their analysis at the hospital level. A key challenge for these studies of the quality of health care is to account for selection and sorting of patients across hospitals. Most studies of health-care quality pay little attention to this issue (e.g. [8-10]). But there are exceptions, and they show that selection issues are empirically relevant in the case of hospital-quality indicators [2,11].

In this paper we exploit an institutional feature of the Norwegian health care system to handle the selection problem. Although cancer patients may receive treatment from more than one hospital, all patients are initially allocated to a local hospital strictly based on their residential address. Therefore, in our empirical analysis, we assign patients to the hospital to which they belong (i.e. to their hospital catchment area) rather than to the hospital(s) they were actually treated at. This approach minimizes the chance that non-random sorting of patients into hospitals bias the estimated quality indicators. At the same time, the interpretation of the quality indicators is slightly different. Observed differences in quality of cancer care may stem from variation in treatment at local hospitals, differences with respect to sending patients to other hospitals with specialized competences, and regional differences in the quality of general practitioners (GP).

Our analysis is related to Kravdal's [12] investigation of regional variation in cancer survival rates in Norway. He documents differences in survival rates across Norwegian regions, even when controlling for a limited set of individual and regional characteristics. We extend upon Kravdal's analysis by utilizing a much richer set of patient characteristics, as well as considering alternative outcome measures.

## Methods

### Institutional setting

All Norwegian citizens are covered by the public health care system. In the period that we study, 1987-2000, the responsibility for health-care services was shared between the regional and local levels of government. Municipalities (n = 440) had (and still have) the responsibility for primary health care (first and foremost provided by GPs), including both preventive and curative treatment. Counties (n = 19) had the responsibility for specialized treatment at psychiatric and somatic hospitals. In 2002, the responsibility for specialized health care services was transferred from the regional to the national government. Free hospital choice was introduced in 2001. For more institutional details, see [13].

Prior to 2001, patients were allocated to hospitals based on their residential address. In 2000, 55 such catchment areas existed. Nine catchment areas have more than one hospital. These catchment areas typically have one main hospital and between one and three smaller units, typically offering specialized treatment of diseases other than cancer.

To receive hospital treatment, except for emergency care, all citizens have to be referred by a GP. Where more specialized or intensive treatment is required, patients are referred to or transferred to hospitals outside their catchment area (either by the local hospital or by the GP). In our data set, hospitals are made anonymous and we cannot therefore offer any evidence on the extent of treatment provided outside catchment areas.

Both somatic hospitals and hospital catchment areas vary considerably in size. The number of patients that each catchment area is responsible for varies from about 13,000 to about 507,000, with an average of about 90,000. In most cases, hospital catchment areas do not span more than one county.

### Data

We use individual level data from The Norwegian Cancer Registry for the period 1987 to 2000. Reporting to the Cancer Registry is mandatory (and done by clinicians and pathologists), and the completeness of registration for solid tumors is close to 100 percent [14,15]. The Cancer Registry includes information on date of diagnosis; location of the tumor (cancer type); characteristics of the tumor; and the date of death (where applicable).

Patients are identified in the Cancer Registry by a unique personal identification number. This allows us to merge in data on socio-economic background characteristics, local government of residence and labor market outcomes from different administrative registers from Statistics Norway.

Our analysis relies on two different criteria to assess the quality of health services: survival and employment, both measured five years after diagnosis. Clearly, employment is not a relevant indicator of wellness for all cancer patients. We exclude patients that are older than 59 years (approaching retirement) and younger than 21 (less likely to have entered the labor force) on the date of diagnosis. More than 70 percent of the total number of cancer patients is excluded from the analysis due to this restriction.

During the period 1987 to 2000, the hospital catchment structure was relatively stable. Most of the changes in the catchment structure were driven by catchment areas being merged (the total number of catchment areas decreased from 63 to 55 from 1987 to 2000). In our analysis, we rely on the catchment area structure that existed in 2000. We drop patients living (at the time of diagnosis) in local governments that did not belong to the same catchment area for the entire period (18 local governments). This implies dropping less than 2 percent of all patients. Our final sample consists of 46,720 cancer patients in 55 hospital catchment areas.

### Empirical approach

Our strategy is to view the outcome of a cancer patient as depending on characteristics of the disease, characteristics of the patient (in addition to the disease) and the hospital to which the patient belongs. In addition, general time trends and random variation may affect the outcomes. In order to obtain estimates of outcome differences across hospital catchment areas, conditional on the composition of the patients, we base our analysis on the following model (cf. [2]):

(1) $${\text{y}}_{\text{ijt}}={\text{Disease}}_{\text{i}}\beta +{\text{Patchar}}_{\text{i}}\alpha +{\text{z}}_{\text{j}}\eta +\theta_{\text{t}}+\varepsilon_{\text{ijt}}$$

Y_ijt _is an outcome measure that takes a value of one if patient *i *who is a resident in hospital catchment area *j *is alive/working five years after being diagnosed with cancer for the first time on date *t*, and zero otherwise. *Disease*_*i *_is a vector consisting of variables describing the characteristics of the diseases such as the cancer type (the most detailed International Classification of Diseases, 7^th ^revision) and the degree of metastasis at the time of diagnosis. *Patchar*_*i *_is a vector containing variables describing the patients' demographic and socio-economic status (age, education level, gender and marital status), but we also include labor market status and industry affiliation (41 dummies) for the year prior to diagnosis. Labor market status before diagnosis is a proxy for unobserved patient characteristics, such as the patients general health status and general attachment to the labor market. For a full description of all included control variables, see Table 1.

**Table 1:** Control variables, summary statistics

	Mean	St.dev
DEMOGRAPHIC CHARACTERISTICS		
Age at the date of diagnosis	47.86	8.94
Female	0.59	0.49

		

SOCIOECONOMIC CHARACTERISTICS		
Education		
*- Lower secondary or less*	0.29	0.46
*- Upper sec. (11-12 years)*	0.30	0.46
*- Upper sec. final (13 years)*	0.15	0.36
*- Upper sec. extension (14 years)*	0.03	0.16
*- Higher ed.- lower level (14-17 years)*	0.18	0.38
*- Higher ed.- upper level (18+ years)*	0.05	0.21
Marital status		
*- Married*	0.67	0.47
*- Never married*	0.16	0.36
*- Widow/widower*	0.03	0.17
*- Divorced*	0.12	0.33
*- Separated*	0.03	0.16
Labor market status the year prior to diagnosis		
*- Not in labor market*	0.23	0.42
*- Employee (part time, 4-19 hours per week)*	0.09	0.28
*- Employee (part time, 20-29 hours per week)*	0.10	0.31
*- Employee (full time, 30 h+ hours per week)*	0.58	0.49
Dummy variables for industry		
*- Agriculture including hunting*	0.0034	0.0582
*- Forestry*	0.0010	0.0317
*- Fishing*	0.0017	0.0408
*- Mining of coal and lignite*	0.00002	0.0046
*- Extraction of crude petroleum and natural gas*	0.0082	0.0902
*- Mining of metal ores\*	0.0007	0.0266
*- Other mining and quarrying*	0.0013	0.0358
*- Manufacture of food products, beverages and tobacco*	0.0214	0.1448
*- Manufacture of textiles (including footwear) and textile products including leather*	0.0047	0.0680
*- Manufacture of wood and wood products*	0.0087	0.0927
*- Wood processing, graphic production and publishers*	0.0173	0.1304
*- Manufacture of chemical, oil, coal, plastic and rubber products*	0.0110	0.1044
*- Manufacture of mineral products*	0.0045	0.0666
*- Manufacture of metal*	0.0084	0.0912
*- Manufacture of tools*	0.0422	0.2011
*- Other manufacturing products*	0.0033	0.0573
*- Electricity and gas supply*	0.0080	0.0890
*- Water supply*	0.0002	0.0146
*- Construction*	0.0386	0.1926
*- Wholesale and agency business*	0.0466	0.2108
*- Retail trade*	0.0582	0.2342
*- Hotels and restaurants*	0.0156	0.1240
*- Transport, storage and communication*	0.0421	0.2009
*- Post and telecommunications*	0.0195	0.1384
*- Financial intermediation*	0.0210	0.1433
*- Insurance and pension funding (except compulsory social security)*	0.0059	0.0768
*- Real estate, renting and business activities*	0.0396	0.1950
*- Public administration and defense*	0.0722	0.2589
*- Waste management and cleaning*	0.0045	0.0669
*- Personal service activities*	0.0072	0.0848
*- Embassy activities (both international and national)*	0.00002	0.0046
*- Education and training activities*	0.0814	0.2735
*- Research activities*	0.0047	0.0680
*- Health and veterinary activities*	0.0920	0.2891
*- Social work activities*	0.0508	0.2197
*- Activities of professional organizations*	0.0044	0.0663
*- Activities of other membership organizations*	0.0039	0.0625
*- Motion picture, video, radio and television activities*	0.0036	0.0600
*- Library, archives and museum activities*	0.0034	0.0579
*- Sporting activities and other recreational and cultural activities*	0.0022	0.0465

N = 46,720

All the variables included in Equation (1) are dummy variables, i.e., variables that can take a value one or zero, e.g., z_j _takes a value one if patient *i *is a resident in hospital catchment area *j*, and zero otherwise. For more information on dummy variables, see Wooldridge [16] p. 211. When all control variables are discrete, as in our case, a linear probability model is appropriate [17]. We consequently estimate Equation (1) using Ordinary Least Squares (OLS). We have also experimented with a logistic specification, and the results are similar.

Our parameters of interest are η. η is a vector of hospital catchment specific effects denoting the effect on Y_ijt _of being a resident of hospital catchment area *j*. As quality of care is measured at the catchment area level, the estimated η will capture differences in the quality of care stemming from differences in local hospital quality, differences in the quality of general practitioners and differences with respect to sending patients to other hospitals with specialized competences. General time trends, common across all catchment areas, are taken out by a vector of year dummies, θ_t_. Finally, ε_ijt _is an error term assumed to be independent and identically distributed. When estimating Equation (1) with a linear probability model, the estimated η can be interpreted as the average success (survival or employment) probability in hospital catchment area *j*, if catchment area *j *were faced with an average disease and patient composition. Similarly, the coefficients for the other dummy variables have simple interpretations as conditional probability differences.

Why is our empirical approach where we assign patients to the hospital they belong to more appropriate than assigning patients to the hospital(s) in which they were actually treated? The latter approach is problematic because one would need to assume that patients that are transferred to hospitals other than their home hospital are similar to other patients (conditional on disease and patient characteristics). If this is not the case, e.g., if patients are transferred from smaller to larger hospitals for more specialized treatment, and these patients suffer from more severe diagnoses (that we do not observe or cannot control for), the results will be biased towards finding well-performing small hospitals and poor-performing larger hospitals. In addition, even though free hospital choice was not introduced in Norway in the period covered by our analysis, one cannot exclude the possibility that some patients had knowledge of which hospitals provided better treatment, and were able to be referred or transferred to these hospitals. Another related methodological challenge is that patients sometimes receive treatment at more than one hospital (approximately 40 percent of the patients in our sample receive treatment at more than one hospital). It is not obvious how one should weight each hospital's contribution to treatment. The most straightforward solution would be to give each hospital equal weight. However, in many cases, this is an unreasonable assumption, e.g. when patients are immediately transferred from one hospital to another, or if patients get transferred from one hospital to another only to receive palliative care.

In general, the sign of the bias is not known when the selection problem is ignored. In a study related to ours, Gowrisankaran and Town [2] find that quality differences across hospitals are magnified when the selection problem is accounted for in a study of pneumonia patients in California. They use the distance from each patient's address to any given hospital as instrumental variables for hospital choice.

Our empirical approach, where we assign patients to the hospital to which they belong minimizes the chances of selection biases, although we cannot rule out that sorting of patients across hospital catchment areas take place. To empirically investigate this possibility, we check whether estimated η are sensitive to the inclusion of our rich set of disease and patient characteristics. If they are insensitive to relevant observable characteristics, they are unlikely to change much if we could control for potentially relevant unobservable characteristics. For a more formal discussion of this argument, see [18].

## Results

### Descriptive statistics

The most common cancer type in our sample is breast cancer (22 percent). The second and third most prevalent cancer types are lung cancer (8 percent) and skin cancer (8 percent).

On average, 65 percent of the cancer patients in our sample were alive five years after diagnosis and 39 percent were employed. However, these figures vary across cancer types. While about 80 percent (50 percent) of patients diagnosed with breast cancer were alive (working) five years after diagnosis, only 13 percent (5 percent) of lung cancer patients were.

In our sample 43 percent of the cancer patients had a localized tumor on the date of diagnosis (i.e., the tumor was located only in the originated tissue). A total of 34 percent had a distant tumor (i.e., the malignant tumor has spread to other lymph nodes or organs). This group can be further divided into two subcategories: patients with regional cancer (which had spread to nearby lymph nodes, 18 percent of the sample) and distant cancer (which had spread to other organs or lymph nodes farther away, 16 percent of the sample). For 24 percent of the patients, specialists were not able to determine degree of metastasis (these patients are therefore reported in the data to have an unknown degree of metastasis on the date of diagnosis). The association between the type of the tumor and the chances of surviving is high. The average five year survival rate is 83 percent for those with a localized tumor, 61 percent for those with regional spread and only 14 percent for those with distant cancer. Patients with localized cancer are also more likely to be employed. Five years after the year of diagnosis, 52 percent of these individuals are employed. The corresponding numbers for patients with regional and distant cancer are 34 percent and 7 percent, respectively.

### Variation in quality of care

In our analysis, we focus both on unconditional estimates, which we refer to as unadjusted indicators, and on estimates based on the full set of control variables that we have available, which we refer to as adjusted indicators. As explained above, the estimated catchment area fixed effects can be considered as indicators of the quality of health care in hospital catchment area j, i.e. the average survival or employment probability in catchment area j if it were faced with an average disease and patient composition. For both adjusted success measures, the F-test rejects the null hypothesis of no regional variation in quality of care (employment p < .01, survival p < .05).

**Figure 1:**
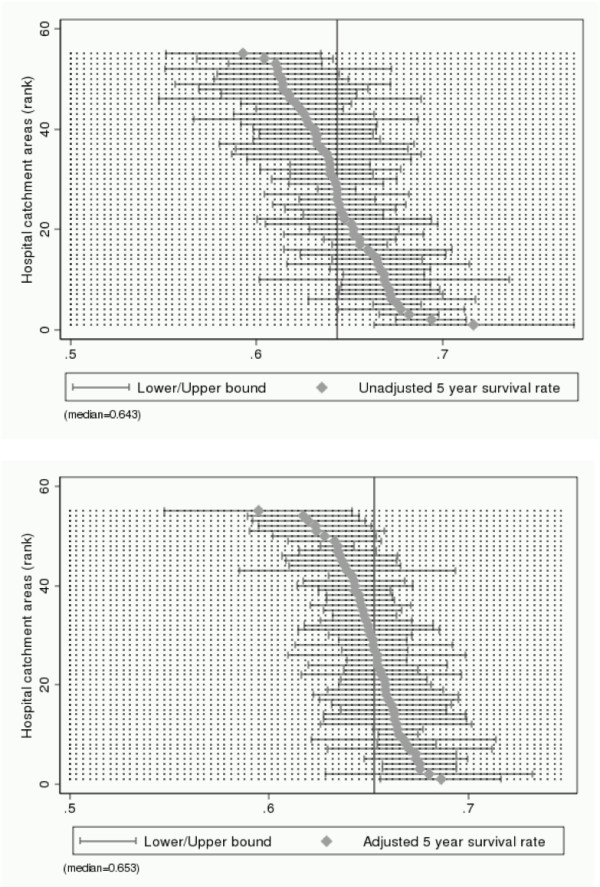
**Indicators of quality of cancer care, based on survival rates and corresponding 90 percent confidence intervals**. Note: Catchment areas are ranked along the vertical axis from the best perfomer (rank #1) to the worst peformer (rank #55). Unadjusted indicators (top figure) are the average survival probability in each catchment area. Adjusted indicators (bottom figure) are interpreted as the average survival probability in each catchment area if it were faced with an average disease and patient composition. Survival is measured five years after diagnosis. The vertical line denotes the median performing hospital catchment area.

The point estimates of quality of care imply that there are differences between hospital catchment areas with respect to both survival and employment after cancer (when holding patient and disease characteristics fixed). However, the quality indicators are estimated with uncertainty which has to be taken into account. The uncertainty related to each point estimate of health care quality is illustrated in Figures 1 (survival) and 2 (employment) which report 90 percent confidence intervals for unadjusted (top figure) and adjusted indicators (bottom figure). For both survival and employment, we find that 12 confidence intervals out of 55 (22 percent) do not contain the point estimate of the median performing catchment area (indicated with the vertical line in the figures). To further clarify to what extent the differences in estimated quality of care are statistically significant, we test all 1485 pair-wise combinations of catchment areas under the null hypothesis of no quality differences. At the ten percent significance level, we find that 15.1 percent of the unadjusted survival rates and 11.7 percent of the adjusted survival rates are significantly different from each other. For employment we find that 39.4 percent of the unadjusted rates and 23.2 percent of the adjusted rates are significantly different from each other.

**Figure 2:**
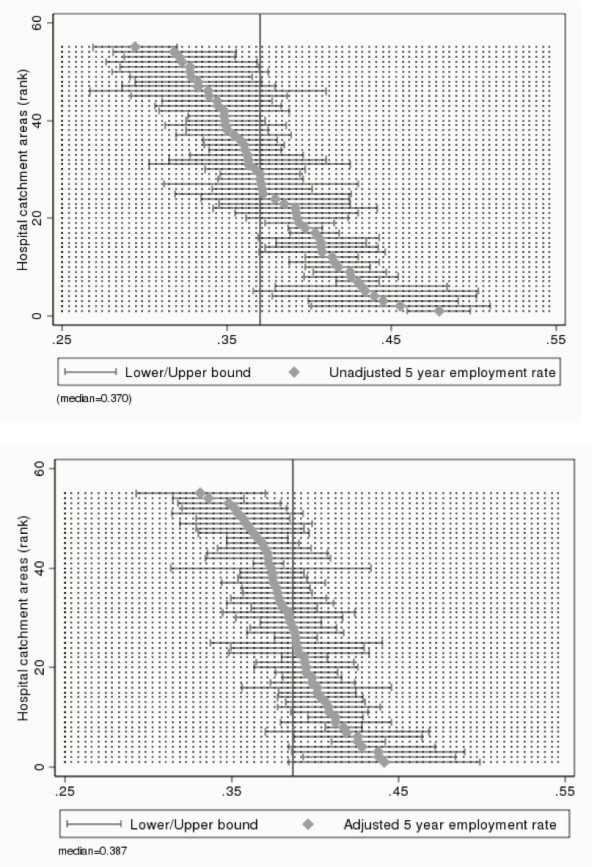
**Indicators of quality of cancer care, based on post-diagnosis employment rates and corresponding 90 percent confidence intervals**. Note: Catchment areas are ranked along the vertical axis from the best perfomer (rank #1) to the worst peformer (rank #55). Unadjusted indicators (top figure) are the average post-diagnosis employment probability in each catchment area. Adjusted indicators (bottom figure) are interpreted as the average post-diagnosis employment probability in each catchment area if it were faced with an average disease and patient composition. Post-diagnosis employment is measured five years after diagnosis. The vertical line denotes the median performing hospital catchment area.

**Figure 3:**
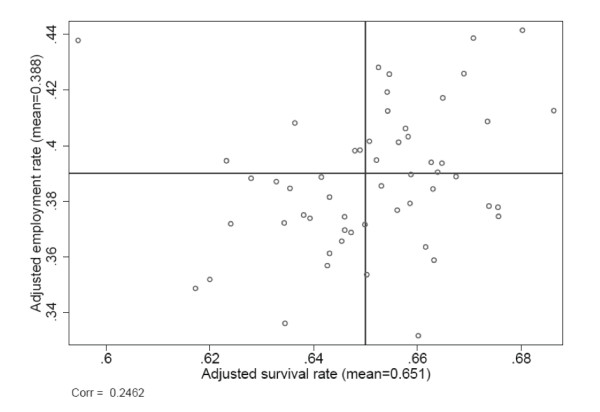
**Relation between quality indicators based on survival and employment rates**. Note: Adjusted indicators are interpreted as the average success probability in each catchment area if it were faced with an average disease and patient composition. Both success measures are measured five years after diagnosis.

The key objective of this study is to compare quality indicators based on post-diagnosis employment to quality indicators based on survival. Central to our analysis is therefore Figure 3, where we document the correlation between the two sets of quality indicators. The correlation coefficient between quality indicators based on survival and post-diagnosis employment is 0.26.

As documented in Tables 2 and 3, we find characteristics of the disease to be the major explanatory factors of survival and employment. Since we employ a linear probability model, the coefficients have straightforward interpretations as the all-else-equal differences in the survival/employment probability between the actual category and the reference category. E.g., in Table 2, column 4, the coefficient 0.0480 for Female implies that women, all else equal, has a survival probability that is 4.80 percent higher than men. Having a metastatic tumor on the date of diagnosis lowers the chances of both survival and employment (with a p-value of less than 0.01, denoted by three asterisks). Demographic and socio-economic variables (e.g., gender, age, level of education and marital status) are also strongly associated with successful treatment. Finally, both survival and post-cancer employment is positively associated with being in the labor market one year prior to diagnosis (p < .01). The industry dummy variables are jointly statistically significant for employment (p < .01) and statistically insignificant for survival.

**Table 2:** The relation between observables and survival five years after diagnosis

	(1)	(2)	(3)	(4)	(5)
Metastasis (Reference: Localized)					
*- Regional*		-0.1826	-0.1815	-0.1792	-0.1787
		(0.0054)***	(0.0054)***	(0.0054)***	(0.0054)***
*- Distant*		-0.5363	-0.5335	-0.5292	-0.5269
		(0.0060)***	(0.0060)***	(0.0059)***	(0.0059)***
*- Unknown*		-0.1151	-0.1144	-0.1120	-0.1098
		(0.0056)***	(0.0056)***	(0.0056)***	(0.0056)***
Female			0.0439	0.0480	0.0547
			(0.0046)***	(0.0047)***	(0.0051)***
Education (Reference: Lower secondary or less)					
*- Upper sec. (11-12)*				0.0224	0.0155
				(0.0045)***	(0.0045)***
*- Upper sec. final (13)*				0.0407	0.0310
				(0.0056)***	(0.0057)***
*- Upper sec. extension (14)*				0.0437	0.0324
				(0.0110)***	(0.0111)***
*- Higher ed.- lower level (14-17)*				0.0507	0.0402
				(0.0053)***	(0.0058)***
*- Higher ed.- upper level (18+)*				0.0744	0.0660
				(0.0087)***	(0.0091)***
Marital status (Reference: Married)					
*- Never married*				-0.0446	-0.0401
				(0.0054)***	(0.0054)***
*- Widow/widower*				-0.0318	-0.0274
				(0.0103)***	(0.0103)***
*- Divorced*				-0.0257	-0.0225
				(0.0053)***	(0.0054)***
*- Separated*				-0.0294	-0.0267
				(0.0110)***	(0.0110)**
*- Other*				-0.0822	-0.0881
				(0.1111)	(0.1110)
Labor market status prior to diagnosis (Reference: Not in labor market)					
*- Employed (part time, 4-19 hours per week)*					0.0611
					(0.0211)***
*- Employed (part time, 20-29 hours per week)*					0.0554
					(0.0209)***
*- Employed (full time, 30+ hours per week)*					0.0569
					(0.0204)***

R^2^	0.0044	0.4031	0.4078	0.4126	0.4154
F-statistic for joint significance of *η*_*j *_	1.81***	1.64***	1.65***	1.36**	1.39**

Note: N = 46,720. N catchment areas = 55. Reported are OLS estimates. Standard errors within brackets. Year dummies and a constant term are included in all specifications. Included in specification (2)-(5) are dummy variables for cancer type. Included in specifications (3)-(5) are dummy variables for age. Included in specifications (4) and (5) are dummy variables for missing information on marital status and education. Included in specification (5) are dummy variables for industry. */**/*** statistically significance at the 10/5/1 percent level respectively.

**Table 3:** The relation between observables and employment five years after diagnosis

	(1)	(2)	(3)	(4)	(5)
Metastasis (Reference: Localized)					
*- Regional*		-0.1366	-0.1368	-0.1317	-0.1294
		(0.0064)***	(0.0063)***	(0.0062)***	(0.0059)***
*- Distant*		-0.3302	-0.3258	-0.3155	-0.3044
		(0.0071)***	(0.0069)***	(0.0068)***	(0.0066)***
*- Unknown*		-0.0756	-0.0741	-0.0686	-0.0578
		(0.0067)***	(0.0066)***	(0.0064)***	(0.0062)***
Female			-0.0219	-0.0050	0.0214
			(0.0054)***	(0.0054)	(0.0056)***
Education (Reference: Lower secondary or less)					
*- Upper sec. (11-12)*				0.0856	0.0469
				(0.0051)***	(0.0050)***
*- Upper sec. final (13)*				0.1479	0.0907
				(0.0065)***	(0.0063)***
*- Upper sec. extension (14)*				0.1534	0.0879
				(0.0126)***	(0.0122)***
*- Higher ed.- lower level (14-17)*				0.2102	0.1251
				(0.0061)***	(0.0064)***
*- Higher ed.- upper level (18+)*				0.2399	0.1574
				(0.0099)***	(0.0100)***
Marital status (Reference: Married)					
*- Never married*				-0.0437	-0.0262
				(0.0062)***	(0.0060)***
*- Widow/widower*				-0.0334	-0.0117
				(0.0118)***	(0.0114)
*- Divorced*				-0.0451	-0.0339
				(0.0061)***	(0.0059)***
*- Separated*				-0.0539	-0.0441
				(0.0126)***	(0.0122)***
*- Other*				-0.2165	-0.2405
				(0.1275)*	(0.1227)*
Labor market status prior to diagnosis (Reference: Not in labor market)					
*- Employee (part time, 4-19 hours per week)*					0.2360
					(0.0233)***
*- Employee (part time, 20-29 hours per week))*					0.2837
					(0.0231)***
- Employee (full time, 30* hours per week))					0.3087
					(0.0225)***

R^2^	0.0038	0.1885	0.2280	0.2566	0.3127
F-statistic for joint significance of *η*_*j*_	5.35***	4.89***	5.05***	3.40***	2.36***

Note: N = 46,720. N catchment areas = 55. Reported are OLS estimates. Standard errors within brackets. Year dummies and a constant term are included in all specifications. Included in specification (2)-(5) are dummy variables for cancer type. Included in specifications (3)-(5) are dummy variables for age. Included in specifications (4) and (5) are dummy variables for missing information on marital status and education. Included in specification (5) are dummy variables for industry. */**/*** statistically significance at the 10/5/1 percent level respectively.

**Figure 4:**
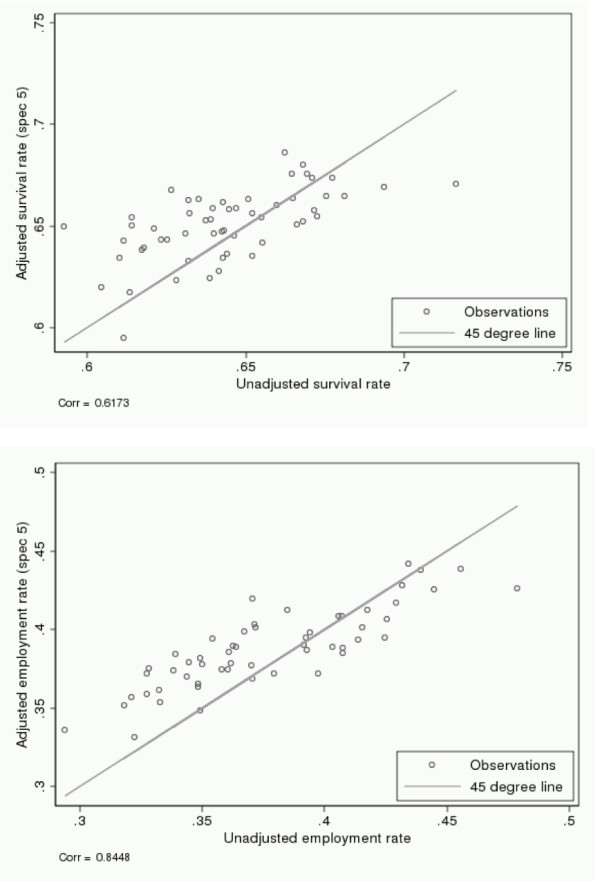
**Relation between unadjusted and adjusted indicators**. Note: Unadjusted indicators are the average success probability in each catchment area. Adjusted indicators are interpreted as the average success probability in each catchment area if it were faced with an average disease and patient composition. Both success measures are measured five years after diagnosis.

In Figure 4, we show scatterplots of unadjusted and adjusted success rates for the two outcomes. The correlation between unadjusted and adjusted indicators is 0.62 for survival and 0.84 for employment. After controlling for characteristics of the disease the estimated catchment area fixed effects are insensitive to the inclusion of additional control variables. The correlation between a disease-only adjusted specification and the fully adjusted specification is 0.97 for survival and 0.91 for employment.

To empirically investigate whether differential trends in local labor market conditions may be biasing our results, we include a control variable capturing changes in the local unemployment rate (measured at the local government level) from year *t *(diagnosis year) to year *t+5 *(five years after diagnosis). The point estimate indicates that the probability of employment decreases if local economic conditions are worsening, but the effect is not statistically significant at conventional levels and results are not reported for brevity. The inclusion of this variable leaves the hospital catchment area fixed effects, as well as the corresponding confidence intervals, basically unaltered.

In our baseline specification, the exclusion criteria are based on age. An alternative is to restrict the sample to those who were employed prior to diagnosis. This yields results qualitatively similar to the ones reported above. The correlation coefficient between the baseline catchment area estimates and those obtained from this specification are 0.97 for employment and 0.84 for survival.

We also estimate Equation (1) separately for patients with a localized and distant tumor (patients regional and distant spread are pooled). For the sample restricted to patients with a localized tumor the null hypothesis of no regional variation in quality of care have to be rejected for both post-diagnosis employment (p < .01) and survival (p < .05).

For the sample restricted to patients with a distant tumor the null hypothesis of no regional variation in quality of care can be rejected for post-diagnosis employment, but only at the ten percent level of confidence. For survival we cannot reject the hypothesis of no regional variation in quality of care (p = 0.13).

When we stratify the population by disease severity we find that the correlation coefficient between quality indicators based on survival and post-diagnosis employment is 0.13 for the sample based on patients with a localized tumor and 0.50 for the sample based on patients with a distant tumor.

## Discussion

In this paper we document that there are regional variation across Norwegian hospital catchment areas in the quality of cancer treatment. For both sets of quality indicators that we use, survival and post-diagnosis employment, we find that 22 percent of the hospital catchment areas have 90 percent confidence interval that do not contain the point estimate of the median performing catchment area. This results is similar to the finding of Gowrisankaran and Town [2], studying hospital performance in California, who report that 28 percent of the hospitals in their sample are significantly better or worse than the average hospital, at the 10% level of confidence.

The main contribution of this paper is to compare quality indicators based on survival to quality indicators based on post-diagnosis employment. In Figure 3, we show that the correlation across the two sets of quality indicators is modest. This strongly suggests that conventional quality indicators based on differences in survival rates may not reveal the full picture of differences in quality of care across units.

For the estimated hospital catchment area fixed effects to be given an interpretation as quality indicators we need to assume that the there is conditional random assignment to hospital catchment areas, i.e. patients are not sorted across hospitals catchment areas based on factors that we do not control for and which simultaneously affect health outcomes.

In the empirical analysis, we rely on a very rich and comprehensive data set, containing patient-level clinical indicators (such as the type of cancer and extent of metastasis) and patient-level demographics (including employment status and occupation prior to diagnosis). We find that severity of the disease, as well as socio-economic background variables are strongly associated with successful outcomes. The latter is in line with previous studies that document considerable variation in health outcomes across socio-economic groups (e.g. [19]).

Our identification strategy hinges on the extent to which these observable characteristics capture relevant information on diagnoses and patient characteristics that may matter for outcomes. Our identifying assumption may fail to hold if, for example, the extent to which patients suffer from co-morbidities or comply with medical protocols, systematically varies across catchment areas, conditional on all control variables. As discussed above, the extent of selection on observables may indicate to what extent selection on unobservables is likely to bias our hospital catchment area fixed effects [18].

Figure 4 shows that unadjusted and adjusted estimates of quality of care reveal broadly the same picture for both survival (top figure) and post-diagnosis employment (bottom figure). Hospital areas that do well according to unadjusted indicators also tend to do well according to the adjusted ones. However, for some catchment areas adjusting for patient characteristics gives substantial new information. In the figures, this is seen as deviations from the 45-degree line. From the correlation coefficients, one might conclude that adding observed characteristics matters more for indicators based on survival than for those based on employment. However, the correlation coefficients do not take into account the uncertainty related to each point estimate.

As the estimates change very little when we include a rich set of observable patient characteristics on top of disease characteristics, they are unlikely to change much if we could include potentially relevant unobserved patient characteristics. Hence, selection on unobservables does not seem to be a major problem, and observed differences across hospital catchment areas are likely to stem from actual differences in quality of care.

Our analysis compares the two indicators for the set of patients where both are relevant - the working-age population. Our main finding is that only using indicators based on survival may be insufficient, and that indicators based on different outcomes may capture quality differences in different parts of the health status distribution. To do the similar analysis for other age groups would require another measure of health status for the whole population. Unfortunately, we do not have such a measure available. Whether our findings may be generalized to the whole population of patients depends critically on the extent to which indicators are invariant with respect to patient and disease characteristics (which will typically vary with the age restrictions of the sample). The data we have available does not permit a comparison between indicators based on survival for the working-age population and the whole population, since (hospital catchment) area of residence is only available for the working-age population.

Our main finding, that indicators based on variation in quality of care substantially differ according to the outcome measures used, is robust to a large number of robustness checks. This result is basically unaltered if we control for secular labor market trends, stratify our analysis by severity of disease or alter the inclusion criteria from being based on age to pre-diagnosis employment status.

For post-diagnosis employment, we find stronger evidence in favor of regional variation in the quality of care for the sample based on patients that do not have metastatic cancer, where expected survival is relatively high. This is reasonable, because when expected survival is relatively low, as with metastatic cancer, employment is probably less relevant as outcome measure as fewer patients are at this margin.

In 2001, there was a substantial reform of the hospital sector, where a key element was to give patients the right to choose which hospital to be treated at. The introduction of free hospital choice may have had two effects. First, patients are less tied to their local hospital, and it may matter less which hospital you belong to. Second, free hospital choice and publishing of quality information may induce competition between hospitals, leading to quality improvements and/or low quality hospitals being driven out of the market. Vrangbæk et al. [20] document that relatively few patients take advantage of the opportunity to choose hospitals. It can therefore be argued that the results that we report are likely to be relevant also today. However, a closer investigation of this conjecture is an important topic for future research.

## Conclusions

We have shown that indicators for the quality of health care are sensitive to the outcome measure that forms the basis for the indicator. Hence, conventional quality indicators based on differences in survival rates may not reveal the full picture of differences in quality of care across units. This should be taken into account when designing accountability systems for health care. Our results show considerable differences between catchment areas, with respect both to the probability of surviving cancer and of being employed after cancer. Although there is uncertainty associated with the estimates, a substantial fraction of the differences is statistically significant. It may actually matter which hospital you belong to. Large differences in outcomes indicate that there may be substantial welfare gains if all institutions adopted best practice. However, our study is silent about what the sources of differences are, a topic for future research.

## Competing interests

The authors declare that they have no competing interests.

## Authors' contributions

JHF and TH conceived the study. MR performed the statistical analysis. All authors contributed in the design of the study, interpretation of the statistical analysis and writing of the manuscript. All authors read and approved the final manuscript.

## Pre-publication history

The pre-publication history for this paper can be accessed here:

http://www.biomedcentral.com/1472-6963/10/204/prepub
